# Comparison of the Effect of CFTR Modulators *elexacaftor*/*tezacaftor*/*ivacaftor* and *lumacaftor*/*ivacaftor* via Serum Human Epididymis Protein 4 Concentration in *p.Phe508del-CFTR* Homozygous Cystic Fibrosis Patients

**DOI:** 10.3390/jcm14176188

**Published:** 2025-09-02

**Authors:** Marianna Pócsi, Libor Fila, Csaba Péterfia, Adrien Halász, Tibor G. Szanto, Beáta Mészáros, Judit Major, István Laki, Hajnalka Szabó, György Panyi, István Balogh, Margarida D. Amaral, Milan Macek Jr., Béla Nagy Jr.

**Affiliations:** 1 Department of Laboratory Medicine, Faculty of Medicine, University of Debrecen, 4032 Debrecen, Hungary; pocsi.marianna@med.unideb.hu; 2 Department of Pulmonology, 2nd Faculty of Medicine, Charles University and Motol University Hospital, 150 06 Prague, Czech Republic; libor.fila@fnmotol.cz; 3 Department of Pediatrics, Faculty of Medicine, University of Pécs, 7624 Pécs, Hungary; peterfia.csaba@pte.hu (C.P.); majorjudit1@gmail.com (J.M.); 4 National Korányi Institute for Pulmonology, 1122 Budapest, Hungary; halaszadr@gmail.com; 5 Department of Biophysics and Cell Biology, Faculty of Medicine, University of Debrecen, 4032 Debrecen, Hungary; szantogt80@gmail.com (T.G.S.); mesz.beata@gmail.com (B.M.); panyi@med.unideb.hu (G.P.); 6 Törökbálint Institute for Pulmonology, 2045 Törökbálint, Hungary; lakiist@gmail.com; 7 Department of Pediatrics, Albert Szent-Györgyi Medical School, University of Szeged, 6725 Szeged, Hungary; szabo.hajnalka@med.u-szeged.hu; 8 Department of Medical Genetics, Faculty of Medicine, University of Debrecen, 4032 Debrecen, Hungary; balogh@med.unideb.hu; 9 BioISI-Biosystems & Integrative Sciences Institute, Faculty of Sciences, University of Lisboa, 1749-016 Lisboa, Portugal; msamaral@ciencias.ulisboa.pt; 10 Department of Biology and Medical Genetics, 2nd Faculty of Medicine, Charles University and Motol University Hospital, 150 06 Prague, Czech Republic; milan.macek.jr@lfmotol.cuni.cz

**Keywords:** cystic fibrosis, HE4, CFTR modulator, ppFEV1, biomarker, treatment efficacy

## Abstract

Elevated human epididymis protein 4 (HE4) levels decreased in patients with CF (pwCF) in response to CFTR-specific drugs and negatively correlated with FEV1% predicted values (ppFEV1). **Objectives:** Although *elexacaftor*/*tezacaftor*/*ivacaftor* (ETI, Kaftrio^®^) demonstrates more substantial effectiveness than *lumacaftor*/*ivacaftor* (LUM/IVA, Orkambi^®^) in pwCF, plasma biomarkers have not been used to compare treatment efficacy. Hence, our aim was to correlate the change in HE4 levels and the clinical effects of these CFTR modulators (CFTRm). **Methods:** Serum HE4 concentrations were measured in a total of 123 pwCF homozygous for the *p.Phe508del-CFTR* variant before treatment and 1–6 months after either ETI or LUM/IVA administration. A correlation between serum HE4 and ppFEV1 was assessed using the Spearman test. HE4 protein levels were also analyzed in the supernatants of *p.Phe508del-CFTR* CFBE 41o- cells before and after treatment with these CFTRm, and their direct effect on CFTR function was monitored by the whole-cell patch-clamp technique. **Results:** Serum HE4 levels were reduced below baseline after 3 months of either ETI or LUM/IVA (mean delta HE4: −38.5 vs. −18.5 pmol/L, respectively) when the mean change of ppFEV1 was 13.6 vs. 1.6% and remained decreased up to 6 months. A significant inverse correlation between HE4 and ppFEV1 was observed in both study cohorts (r = −0.537 and r = −0.575, respectively; *p* < 0.0001). In agreement with ex vivo results, the effect on *p.Phe508del-CFTR* was more pronounced by ETI than LUM/IVA in CFBE cells, showing a larger improvement in *p.Phe508del-CFTR* function and reductions in HE4 levels at 24 h. **Conclusions:** Serum HE4 negatively correlates with lung function improvement and monitors better drug efficacy in pwCF under ETI than LUM/IVA.

## 1. Introduction

Cystic fibrosis (CF; MIM: 219700) is a hereditary disorder transmitted in an autosomal recessive pattern, resulting from mutations in the gene encoding the CFTR (cystic fibrosis transmembrane conductance regulator) protein. This protein operates as an epithelial anion channel, facilitating chloride and bicarbonate movement and modulating the hydration and viscosity of secretions in various tissues [1]. Among the more than 2100 identified variants in the *CFTR* gene (MIM: 602421), the *p.Phe508del-CFTR* mutation predominates in Europe, accounting for roughly 80% of alleles responsible for disease manifestation [2]. When CFTR function is compromised, multiple organ systems become affected. In the lungs, deficient chloride transport leads to dehydrated mucosal surfaces and the accumulation of thick mucus, which fosters airway obstruction and persistent infections, commonly with pathogens such as *Pseudomonas aeruginosa*. This cascade of events gradually undermines pulmonary function. Concurrently, pancreatic insufficiency and other systemic complications are common clinical features [1].

In the past decade, treatment strategies have shifted from symptom control to correcting the underlying molecular defect. This shift was enabled by the development of small-molecule drugs that enhance or restore CFTR activity in patients with particular genetic profiles [3]. *Ivacaftor* acts as a potentiator, enhancing gating at the cell surface [4]. In parallel, *lumacaftor* and *tezacaftor* serve as correctors, stabilizing the folding of the *p.Phe508del-CFTR* protein and promoting its cellular trafficking [5,6]. More recently, *elexacaftor* has emerged as an additional corrector, binding at a distinct site to further augment CFTR activity when used in combination [7]. The triple combination of *elexacaftor*, *tezacaftor*, and *ivacaftor* (ETI) has demonstrated clinically meaningful improvements. Data from early-phase trials showed significant increases in ppFEV1: +13.8% after 4 weeks and +14.3% at 24 weeks. Other benefits included a 63% reduction in pulmonary exacerbations and substantial decreases in sweat chloride levels (−45.1 mmol/L) [8,9]. The U.S. Food and Drug Administration (FDA) approved ETI in 2019 for individuals aged 12 or older carrying at least one copy of the *p.Phe508del*-*CFTR* variant. This approval was later extended in 2023 to children aged 2 and above. Follow-up studies have confirmed the treatment’s safety and efficacy in younger cohorts [10,11,12]. For instance, in one trial involving pediatric patients, those on ETI showed a mean reduction of 2.29 units in lung clearance index (LCI2.5), compared to 0.02 in placebo recipients. Sweat chloride levels dropped by an average of 51.2 mmol/L relative to controls, while ppFEV1 improved by 11% over 24 weeks [12].

There is a worldwide trend to administer ETI (Trikafta^®^/Kaftrio^®^) instead of dual-combination LUM/IVA (Orkambi^®^) due to clinical experience that no change or only modest improvements in ppFEV1 and sweat chloride (i.e., −0.3% and −18.5 mmol/L at 12 months, respectively; +2.5% and −24.8 mmol/L by week 24, respectively) could be previously observed in different patient cohorts with two copies of the *p.Phe508del-CFTR* variant under LUM/IVA [13,14].

Recently, we systematically analyzed the level of human epididymis protein 4 (HE4) as a new laboratory biomarker in blood samples of pwCF [15,16,17] and investigated its abnormal expression in CF in vitro [18]. Elevated serum HE4 concentrations were positively associated with the degree of pulmonary dysfunction and overall disease severity of CF in unrelated pwCF cohorts [15], while plasma HE4 levels inversely correlated with lung function improvement in pwCF, showing at least one G551D-CFTR variant receiving IVA [16]. Furthermore, in response to in vitro rescue of CFTR function by LUM/IVA, HE4 expression was lowered in cystic fibrosis bronchial epithelial cell (CFBE) 41o- cells expressing *p.Phe508del-CFTR* [18], while delta HE4 independently indicated the likelihood of being a responder with ≥5% delta ppFEV1 at 6 months in pwCF undergoing LUM/IVA treatment [17].

Here, we further evaluated the utility of HE4 as a reliable biomarker for CF by investigating this protein in subjects receiving ETI therapy. For this purpose, two independent cohorts of pwCF homozygous for *p.Phe508del-CFTR* on ETI were examined for serum levels of HE4. We also aimed to compare the clinical effect of CFTR modulators (CFTRm) Kaftrio^®^ or Orkambi^®^ via altered serum HE4 concentration. In addition, we wanted to confirm our previous findings on the inverse correlation between delta HE4 levels and ppFEV1 values in subjects under ETI medication. Finally, altered HE4 expression in response to CFTRm was modelled in vitro in CFBE cell cultures to validate our ex vivo results.

## 2. Materials and Methods

### 2.1. Clinical Study

In total, 123 pwCF with two *p.Phe508del-CFTR* variants were enrolled from one Czech and four Hungarian CF centers. A total of 80 pwCF aged 6 years or older were treated with Kaftrio^®^, while 43 pwCF were under Orkambi^®^ medication (Vertex Pharmaceuticals, Boston, MA, USA) (Table 1). All study participants were diagnosed per the CF consensus diagnostic criteria [1] and exhibited the classic form of the disease. Evaluation of lung function by spirometry parameter ppFEV1 was calculated based on the current guidelines before and under treatment [19], and sweat chloride concentrations were assessed by sweat conductivity testing before the initiation of CFTRm. Importantly, these analyses were performed on the same day blood samples were drawn.

### 2.2. Study Population and Design

There were two independent cohorts with pwCF on Kaftrio^®^: In (i) (adult) cohort 1 (n = 51) from Prague (Czech Republic), 28 females (54.9%) and 23 males (45.1%) were involved, and the median age was 27 years (min, 20 years; max, 47 years). In this subgroup, two study visits were performed—at baseline and at 3 months after the initiation of ETI—and no further follow-up samples were available due to the outbreak of the COVID-19 pandemic. The second cohort was (ii) cohort 2 (n = 29) from 4 Hungarian CF centers (Pécs, Budapest, Törökbálint, and Szeged), consisting of 17 females (58.6%) and 12 males (41.4%), and the median age was 12 years (min, 6 years; max, 50 years). In this study group, samples were obtained at baseline and 1–2 and 3–6 months after the initiation of therapy. In cohort 1, baseline median ppFEV1 was 73% (24–107), and sweat chloride levels were over 60 mmol/L in all pwCF, with a median (min–max) of 103 mmol/L (61–119). Pre-treatment BMI values (median, 21.9 kg/m^2^) were within the range (min–max) of 17.0–34.4 kg/m^2^. In cohort 2, the baseline lung function parameter was relatively high, with a median ppFEV1 of 89% (40–147) and abnormal sweat chloride levels (median (min–max), 112 mmol/L (66–149)), while pre-treatment BMI values (median, 16.3 kg/m^2^) were within the range (min–max) of 13.2–25.3 kg/m^2^.

To compare the effect of Kaftrio^®^ in contrast to that of Orkambi^®^, 43 age- and gender-matched pwCF on LUM/IVA treatment were also recruited from the Hungarian CF centers mentioned above, and the median age was 14 years (min, 11 years; max, 53 years), with three blood sampling time points, similar to cohort 2 on ETI. In the LUM/IVA cohort, baseline lung function was impaired in most patients, with a median ppFEV1 of 70% (23–120) and elevated sweat chloride levels (median (min–max), 128 mmol/L (61–161)), while pre-treatment BMI values (median, 17.9 kg/m^2^) were within the range (min–max) of 13.2–27.9 kg/m^2^ (Table 1). In all patients, the degree of systemic inflammation was evaluated by C-reactive protein (CRP). Kidney function was monitored by serum creatinine level to exclude the potential influence of impaired renal function on the serum HE4 level [20]. Serum specimens from all recruited pwCF were stored to measure HE4 levels retrospectively.

Furthermore, study participants on Kaftrio^®^ were sub-grouped as (1) “responders” if they experienced an increase in ppFEV1 equal to or larger than 5% at 3 months in cohort 1 (46 of 51, 90.2%) or 6 months in cohort 2 (19 of 29, 65.5%) and (2) “non-responders” if they showed a ppFEV1 value of less than 5% (5 of 51 (9.8%) in cohort 1; 10 of 29 (34.5%) in cohort 2). In the subgroup of pwCF on Orkambi^®^, the ratio of “responders” to “non-responders” was 22 (51.2%) to 21 (48.8%) (Table 1).

### 2.3. Serum HE4 Measurement

Blood samples were obtained through venous puncture, centrifuged for serum, and stored at −70 °C. Aliquots were transferred from Prague and other national CF centers on dry ice by courier service to the University of Debrecen (Hungary). Serum HE4 levels were analyzed using a chemiluminescent microparticle immunoassay (Architect i1000SR^®^, Abbott Diagnostics, Wiesbaden, Germany), as formerly done in other clinical CF studies [15,16,17,18].

### 2.4. Clinical Correlation Analysis

Serum HE4 levels were correlated with ppFEV1. The Spearman correlation coefficient (r) was used to explore relationships between absolute or delta values of serum HE4 and absolute or delta ppFEV1.

### 2.5. In Vitro Experiments

Altered HE4 expression in response to CFTRm was modelled in vitro in cell cultures to validate our ex vivo results. For this purpose, CFBE 41o- cell cultures stably expressing *p.Phe508del-CFTR* or wt-CFTR were applied.

### 2.6. Cell Culture and Treatment with CFTR Modulators

CFBE 41o- cell cultures expressing *p.Phe508del-CFTR* or wt-CFTR were grown in Minimum Essential Medium Eagle (EMEM) with Earle’s BSS (EBSS), 1% L-glutamine (Lonza, Walkersville, MD, USA), 10% fetal bovine serum (FBS, Sigma-Aldrich), and 5 μg/mL puromycin (Sigma-Aldrich) at 37 °C and under 5% CO_2_, as we previously set [18]. These cells were formerly obtained from Dr. J. P. Clancy’s lab (Cincinnati Children’s Hospital Medical Center, OH, USA). CFBE cells were seeded in 6-well plates (250.000 cells per well/sample). For the activation of the CFTR function, Forskolin (FSK) (10 μM) with IBMX (3-isobutyl-1-methylxanthine, 100 μM) (FSK/IBMX) was added to both genotypes of CFBE cells with or without CFTRm, while CFTR inhibition was carried out by CFTR_inh172_ (20 μM) in wt-CFTR CFBE cells vs. control samples with DMSO for 24 h. CFTR modulators were applied under similar experimental conditions as in previous comparable in vitro studies [18,21,22].

CFTR correctors *elexacaftor* (VX-445) (S8851), *lumacaftor* (VX-809) (S1565), and *tezacaftor* (VX-661) (S7059); CFTR potentiator *ivacaftor* (VX-770) (S1144); voltage-independent selective CFTR inhibitor CFTR_inh172_ (S7139); and CFTR activator (FSK, S2449) were purchased from Selleck Chemicals (Houston, TX, USA). cAMP phosphodiesterase inhibitor IBMX (I5879) was ordered from Sigma-Aldrich (St. Louis, MO, USA). All reagents were dissolved in DMSO (Sigma-Aldrich).

### 2.7. HE4 Protein Analysis in Cell Supernatants

Supernatants were collected for the analysis of HE4 protein levels after CFBE cells were treated with combined CFTRm drugs for 24 h: corrector *lumacaftor* (3 μM) with potentiator *ivacaftor* (10 μM) (Orkambi^®^) or correctors *elexacaftor* (3 μM) and *tezacaftor* (5 μM) with *ivacaftor* (10 μM) (Kaftrio^®^) or dimethyl sulfoxide (DMSO) vehicle alone (representing the baseline).

### 2.8. CFTR Functional Assessment (Whole-Cell Patch Clamp)

As in previous publications, Cl^−^ currents in CFBE 41o- cells were measured using the conventional whole-cell patch-clamp configuration [18,23,24]. The external (bath) solution contained 145 mM NaCl, 4 mM CsCl, 1 mM CaCl_2_, 1 mM MgCl_2_, 5 mM D-glucose, and 10 mM HEPES (pH 7.4 titrated with NaOH, 315 mOsm). The intracellular (pipette) solution contained 113 mM L-aspartic acid, 113 mM CsOH, 27 mM CsCl, 1 mM NaCl, 1 mM MgCl_2_, 1 mM ethylene glycol tetra-acetic acid (EGTA), 10 mM HEPES, and 3 mM Mg-ATP (pH 7.2, titrated with CsOH, 285 mOsm). Mg-ATP was freshly diluted in the intracellular solution from 200 mM stock solutions every hour. The intracellular solution was stored on ice before usage. FSK/IBMX and CFTR_inh172_ were diluted from 10 mM stock solutions freshly prepared in water-free DMSO into the extracellular solution before the start of the experiments. Stock solutions were prepared in DMSO.

Bath perfusion around the measured cell with different external solutions was achieved using a gravity-flow micro perfusion system at a rate of 200 μL/min, and the excess fluid was removed continuously. Solutions containing CFTR_inh172_ (Sigma-Aldrich) were also made fresh in the external solution from 25 mM stocks stored at −20 °C. The robust change in the current amplitude upon perfusion with CFTR_inh172_ was an indicator of both the expression of CFTR channels and the proper operation of the perfusion system.

Micropipettes were pulled in four stages using a P-2000 automatic pipette puller (Sutter Instruments, San Rafael, CA, USA) from GC150F-7.5 borosilicate capillaries (GC150-TF10, Harvard Apparatus Co., Holliston, MA, USA) with a tip resistance typically ranging from 3 to 10 MΩ in the bath solution. Cl^−^ currents were recorded using an Axopatch 200B amplifier connected to a personal computer using Axon Digidata 1550A data acquisition hardware (Molecular Devices Inc., Sunnyvale, CA, USA). The holding potential was maintained at –40 mV throughout the experiment, and the cutoff threshold for acceptable series resistance values was 15 MOhm. To monitor the current evolution under drug applications and to confirm the absence of significant leak current, single depolarization pulses from –40 to 0 mV for 1 s were applied every 5 s for 4–5 min. The current–voltage (I–V) relationship was determined by 800 ms long pulses from the holding potential to test potentials between 0 and +60 mV in 20 mV increments every 10 s. Experiments were conducted at room temperature (RT, between 20 and 24 °C). Data were analyzed using the pClamp10.5 software package (Molecular Devices Inc.). Before analysis, current traces were digitally filtered with a three-point boxcar smoothing filter. Before analysis, current traces were corrected for ohmic leak.

### 2.9. Statistical Analyses

Data are expressed as mean with 95% confidence interval (95% CI) or as median (min to max) where indicated. The Shapiro–Wilk and Kolmogorov–Smirnov normality tests were used to evaluate the normality of the data. Due to the non-parametric distribution of clinical and laboratory values, the Mann–Whitney U test was used to compare data between two groups. At the same time, the Wilcoxon matched-pairs signed rank test was applied when results in baseline and follow-up samples were compared. The area under the value of the receiver operating characteristic curve (ROC-AUC) was determined for both absolute and delta HE4 to indicate improved lung function under Kaftrio^®^ or Orkambi^®^ treatment at 3 or 6 months (depending on the subcohorts) when 4% of the mean change of ppFEV1 was set as a binary classifier. The maximum of Youden index (sensitivity, 100-specificity) was determined to identify the cut-off values. A *p* ≤ 0.05 probability level was regarded as statistically significant. Analyses were performed using GraphPad Prism, version 9 (GraphPad Software, La Jolla, CA, USA), and SPSS Statistics software, version 26.0 (IBM Corporation, Armonk, NY, USA).

## 3. Results

### 3.1. Treatment with ETI or LUM/IVA Lowers Serum HE4 Levels in CF Subjects Homozygous for p.Phe508del-CFTR Variant

**Figure 1:**
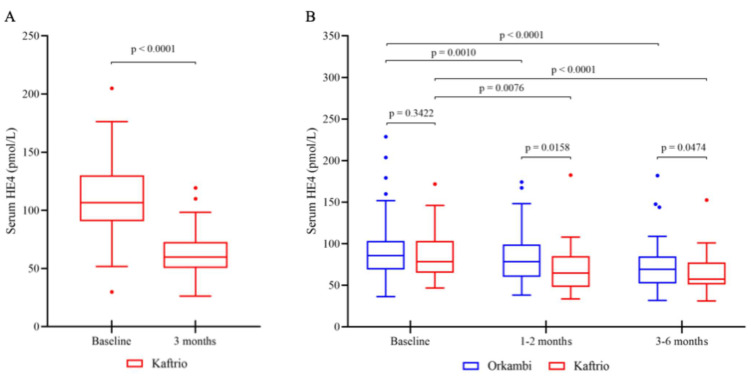
Serum HE4 levels measured at baseline and different follow-up time points under Kaftrio^®^ in cohort 1 (**A**) and Kaftrio^®^ or Orkambi^®^ treatment in cohort 2 or control cohort (**B**) in a total of 123 pwCF homozygous for the *p.Phe508del-CFTR* variant.

First, we determined serum HE4 levels in two independent cohorts with pwCF homozygous for the *p.Phe508del-CFTR* variant who were under Kaftrio^®^ medication. In the presence of substantially improving lung function (17.5 [14.5–20.5]% of ppFEV_1_) in CF adults of cohort 1, there were significantly (*p* < 0.0001) reduced HE4 serum concentrations—regardless of its baseline value—at 3 months of treatment, this being the only follow-up time point of these patients (Figure 1A).

In cohort 2, pulmonary status also improved in most pwCF, showing a mean value of 4.0 [−3.9–12.1]% of ppFEV_1_, and serum HE4 was gradually decreased already at 1–2 months of Kaftrio^®^ treatment (*p* = 0.0076), while further reduction was observed by 3–6 months of therapy (*p* < 0.0001 compared to baseline) (Figure 1B). In parallel, pwCF on Orkambi^®^ also demonstrated significantly lowering serum HE4 values at consecutive visits (*p* = 0.0010 by 1–2 months and *p* < 0.0001 by 3–6 months vs. pretreatment levels). Very importantly, there was no difference in the baseline level of HE4 between the cohorts on Kaftrio^®^ and Orkambi^®^ (*p* = 0.3422). At the same time, HE4 concentrations were significantly lower at each follow-up time point (*p* = 0.0158 at 1–2 months and *p* = 0.0474 at 3–6 months of therapy) in those on ETI compared to those on LUM/IVA, in correlation with the degree of lung function improvement (Figure 1B).

**Figure 2:**
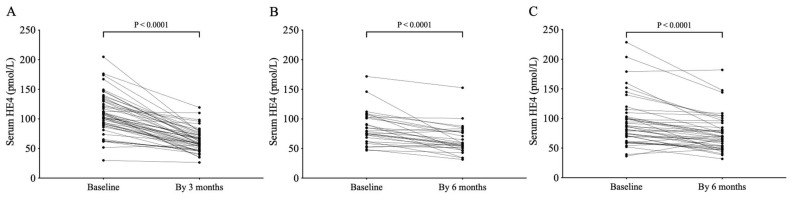
Analysis of individual change in HE4 serum concentrations in cohort 1 (**A**) and cohort 2 (**B**) on Kaftrio^®^, as well as Orkambi-treated control patients (**C**). Wilcoxon matched-pairs signed rank test was applied to determine the difference in HE4 values between baseline and follow-up samples.

Individual alteration in serum HE4 was separately analyzed and was found to be decreased in most study participants observed between the baseline and their last available follow-up time point. In cohort 1, serum HE4 levels were substantially reduced below baseline after 3 months of ETI, with a mean delta HE4 of −45.7 pmol/L when the mean change of ppFEV1 was 17.5%, while ETI pwCF in cohort 2 showed a modest mean value of 4.0% in ppFEV1 by 3–6 months of treatment in the presence of a mean delta value of −20.7 pmol/L HE4 (Figure 2A,B). In contrast, only 1.6% of ppFEV1 was measured in those on Orkambi^®^ medication, with a mean change of HE4 of −18.5 pmol/L by the 3–6-month visit (Figure 2C). These clinical data underscore the impact of CFTRm therapies in terms of decreasing serum HE4 concentrations in CF. Furthermore, the superior therapeutic effect of Kaftrio^®^ over Orkambi^®^ on pulmonary clinical status could be successfully monitored via serum HE4.

### 3.2. HE4 Serum Levels Strongly Correlate with ppFEV1

**Figure 3:**
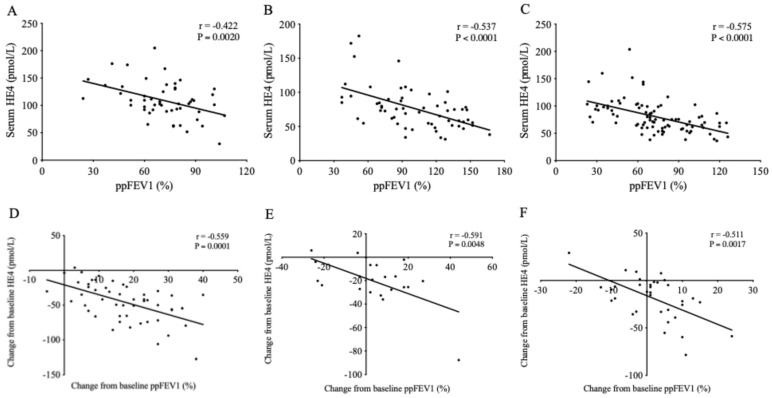
Analysis of the correlation between HE4 serum concentrations and ppFEV1 values in cohort 1 (**A**) and cohort 2 (**B**) on Kaftrio^®^, as well as Orkambi-treated control patients (**C**). The relationship between the mean change in HE4 and delta ppFEV1 was also determined in each study subgroup (**D**–**F**). The Spearman test studied all correlations.

We statistically correlated the HE4 levels with the ppFEV1 values determined on the same day when blood sampling occurred to observe if absolute values of serum HE4 reflected lung function status in each study subgroup. A significant inverse correlation was detected between HE4 and ppFEV1 in all cohorts (r = −0.422, *p* = 0.0020 in cohort 1; r = −0.537, *p* < 0.0001 in cohort 2; and r = −0.575, *p* < 0.0001 in the Orkambi^®^ cohort) (Figure 3A–C).

In parallel, the mean change in HE4 from baseline (i.e., delta HE4) similarly correlated with delta ppFEV1 (r = −0.559, *p* = 0.0001 in cohort 1; r = −0.591, *p* = 0.0048 in cohort 2; and r = −0.511, *p* = 0.0017 in the LUM/IVA cohort) in all study participants (Figure 3D–F). Serum HE4 strongly correlated with ppFEV1 values; thus, it was likely related to CFTRm-induced lung function improvement. Delta HE4 values were even more reflective of ppFEV1 alterations, especially in cohort 2, consisting of relatively young pwCF on ETI treatment.

### 3.3. Change in Serum HE4 Levels Predicts the Improvement of CF Lung Disease Under Kaftrio^®^ Therapy

**Figure 4:**
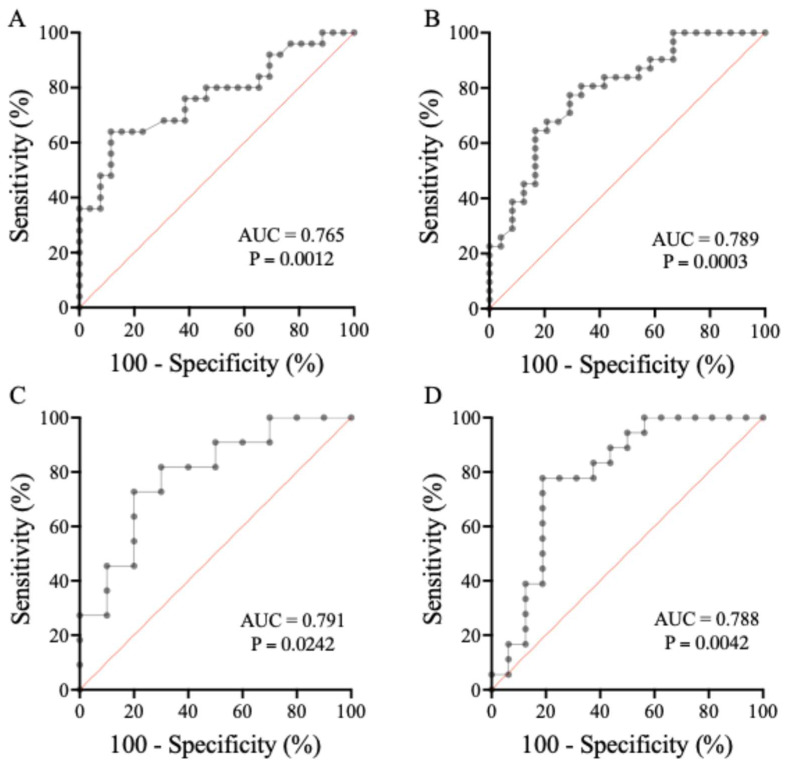
Determination of the discriminatory power of mean change in HE4 if 4.0% of delta ppFEV1 at 3 or 6 months of Kaftrio^®^ treatment was used as the binary classifier using ROC-AUC curve analyses. The mean change in HE4 demonstrated a substantial AUC value in cohort 1 (**A**) and cohort 2 (**B**) in children (**C**) and adults (**D**) on ETI.

We next investigated whether decreasing HE4 levels in response to Kaftrio^®^ were associated with improving CF lung function as assessed by ppFEV1 values. Thus, we calculated the discriminative power of the mean change in HE4 if 4.0% of delta ppFEV1 by 3 or 6 months of treatment was used as the binary classifier in ETI cohorts 1 and 2 and in two age groups of all patients on Kaftrio^®^. A considerable AUC value of delta HE4 (0.765 [95% CI 0.633–0.898], *p* = 0.0012) was found in adult cohort 1, with a cut-off value of −42.4 pmol/L, showing 68.0% sensitivity and 69.0% specificity (Figure 4A), while in cohort 2, with younger individuals, a similarly high AUC value (0.789 [95% CI 0.668–0.909], *p* = 0.0003) was determined in the presence of its cut-off value of −20.3 pmol/L, showing 77.4% sensitivity and 70.8% specificity (Figure 4B). In parallel, Orkambi^®^-treated pwCF showed an AUC value of delta HE4 of 0.809 ([95% CI 0.648–0.969], *p* = 0.0023) when the cut-off value was −17.2 pmol/L, with 78.9% sensitivity and 80.0% specificity (Supplementary Figure S1A). Among the Kaftrio^®^ -treated subjects, the mean change in HE4 was similarly practical in both children (0.791 [95% CI 0.594–0.988], *p* = 0.0242) and adults (0.788 [95% CI 0.624–0.953], *p* = 0.0042) using cut-off values of −14.6 and −21.6 pmol/L, respectively, with sensitivity of 72.7% and 80.0% and specificity of 77.8% and 81.3%, respectively (Figure 4C,D).

We also analyzed the diagnostic characteristics of baseline absolute HE4 levels to predict a satisfactory clinical response to CFTRm with improved lung function of delta 4.0% ppFEV1 by 3–6 months of treatment in any of these cohorts, but no statistically significant relationship was observed under either the Kaftrio^®^ (AUC value of 0.618 [95% CI 0.365–0.872], *p* = 0.3600) or Orkambi^®^ therapy (AUC value of 0.698 [95% CI 0.513–0.883], *p* = 0.050) (Supplementary Figure S1B,C). Hence, delta—but not absolute—HE4 can be effectively applied to indicate the beneficial effects of Kaftrio^®^.

### 3.4. Comparison of the Effects of the Two Different CFTRm on p.Phe508del-CFTR Cl- Currents in CFBE Cells

**Figure 5:**
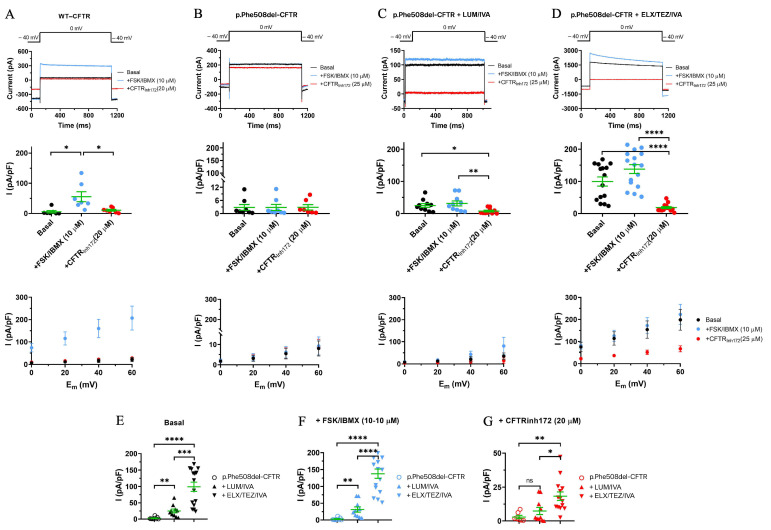
Rescue of functional *p.Phe508del-CFTR* Cl− currents in Kaftrio^®^- or Orkambi^®^-treated CFBE 41o- cells. Representative whole-cell Cl^−^ current traces were elicited by voltage steps from a holding potential of −40 mV to a series of test potentials ranging from 0 to +60 mV in 20 mV increments every 10 s in CFBE 41o- cells expressing wt-CFTR (column **A**) or *p.Phe508del-CFTR* (column **B**) or in cells expressing deletion mutant CFTR but treated for 24 h with LUM/IVA (column **C**) or ELX/TEZ/IVA (column **D**) for 24 h. The duration of the depolarizing pulses was 1 s. For clarity, Cl^−^ currents recorded at 0 mV are shown throughout the figure. *Top panels*: basal currents recorded at 0 mV in the absence of FSK/IBMX stimulation (black), recorded upon ∼2 min stimulation by FSK/IBMX (10/100 μM) (blue) and in the presence of FSK/IBMX and 20 μM of CFTR_inh172_ (red). The *middle panels* show the corresponding peak current densities determined at 0 mV (pA/pF, mean ± SEM) obtained in the absence of FSK/IBMX (basal, black symbols), upon stimulation by FSK/IBMX (blue symbols), and in the presence of FSK/IBMX and 20 μM CFTR_inh172_ (red symbols). The current density for a particular cell was determined as the average of current density values obtained for three to four consecutive depolarizing pulses repeated every 5 s. Bottom panels: current density–voltage relationships (pA/pF, mean ± SEM) measured at the indicated test potentials in the absence of FSK/IBMX (basal, black symbols), upon stimulation by FSK/IBMX (blue symbols), and in the presence of FSK/IBMX and 20 μM CFTR_inh172_ (red symbols). (**E**) Analysis of the basal peak current densities (pA/pF) determined at +40 mV and recorded in cells in the absence of CFTRm pretreatment (F580del, empty circles), pre-treated with LUM/IVA (up triangles) or ELX/TEZ/IVA (down triangles). (**F**) Analysis of the FSK (10 µM)/IBMX (100 µM)-stimulated peak current densities (pA/pF) recorded in cells in the absence of CFTRm pretreatment (*p.Phe508del-CFTR*, empty circles), pre-treated with LUM/IVA (up triangles) or ELX/TEZ/IVA (down triangles). (**G**) Analysis of the FSK/IBMX-stimulated and CFTR_inh172_ (20 µM)-inhibited peak current densities (pA/pF) recorded in cells in the absence of CFTRm pretreatment (F580del, empty circles), pre-treated with LUM/IVA (up triangles) or ELX/TEZ/IVA (down triangles). Data are expressed as mean ± SEM (n = 7–16 cells/condition) unless otherwise indicated, and each symbol represents an individual record. An unpaired or paired t-test was performed for comparisons. Differences were considered significant at *p* < 0.05 (*), *p* < 0.01 (**), *p* < 0.001 (***), and *p* < 0.0001 (****).

The rescue of the CFTR function by CFTRm treatment was monitored using the whole-cell configuration of the patch-clamp technique in airway epithelial cells. Human CFBE 41o- cell cultures expressing *p.Phe508del-CFTR* were treated with two different combinations of CFTR modulators (LUM/IVA or TEZ/IVA) in vitro [18]. In this study, we compared the effects of Kaftrio^®^ and Orkambi^®^ treatment on Cl^−^ channel function using the same methodology when *p.Phe508del-CFTR* expressing CFBE cells were studied with or without ELX/TEZ/IVA or LUM/IVA pretreatment for 24 h (Figure 5B–D). In parallel, untreated wt-CFTR-expressing CFBE cells were analyzed as controls (Figure 5A). One-second-long voltage pulses from the holding potential to 0 mV were applied in the absence of FSK/IBMX to measure the basal Cl^−^ current, in the presence of CFTR activator FSK/IBMX, or when FSK/IBMX treatment was followed by perfusion of the cells with CFTR-selective inhibitor CFTR_inh172_. For easier comparison, the Cl^−^ current amplitude was normalized to the cell capacitance to obtain the current density value, which, for a particular cell, was defined as the average of current densities obtained for at least three depolarizing pulses repeated at every 5 s in a sequence. FSK/IBMX perfusion elicited a robust, CFTR_inh172_-sensitive whole-cell Cl^−^ current (Figure 5A, top and middle panels) with a linear and CFTR_inh172_-sensitive current density–voltage relationship (Figure 5A, bottom panel) in cells expressing wt-CFTR. Perfusion of FSK/IBMX failed to elicit the Cl^−^ current in CFBE cells expressing *p.Phe508del*-CFTR channels (Figure 5B,F). Importantly, pretreatment of *p.Phe508del*-CFTR-expressing cells with either a combination of CFTR modulators (LUM/IVA (Figure 5C) or ELX/TEZ/IVA (Figure 5D)) significantly increased the basal Cl^−^ current (Figure 5E). Moreover, the basal current was significantly larger upon ELX/TEZ/IVA pretreatment as compared to LUM/IVA pretreatment; current densities were (in pA/pF) 2.8 ± 1.3 (n = 8), 24.9 ± 5.8 (n = 10, *p* = 0.0044), and 99.4 ± 14.2 (n = 15, *p* < 0.0001) for untreated *p.Phe508del*-CFTR cells, cells pre-treated with LUM/IVA, and cells pretreated with ELX/TEZ/IVA, respectively. FSK/IBMX stimulated the Cl^−^ current in both LUM/IVA- and ELX/TEZ/IVA-pretreated *p.Phe508del*-CFTR-expressing cells (Figure 5C and Figure 5D, respectively). The FSK/IBMX-stimulated currents were significantly larger compared to the Cl^−^ current measured in the absence of CFTRm pretreatment (Figure 5F). Moreover, the currents were significantly larger upon ELX/TEZ/IVA pretreatment as compared to LUM/IVA pretreatment (138.0 ± 13.8 (n = 16) vs. 31.3 ± 7.7 (n = 10) pA/pF; *p* < 0.0001). The Cl^−^ currents were sensitive to CFTR_inh172_ (Figure 5B–D,G), the FSK/IBMX-induced currents were reduced by 20 μM CFTR_inh172_, to ~10% of the currents measured in the absence of the inhibitor. In summary both CFTRm pretreatments successfully corrected *p.Phe508del-CFTR* function; however, pretreatment with Kaftrio^®^ was more effective than Orkambi^®^, as expected.

### 3.5. ELX/TEZ/IVA Treatment Reduced HE4 Concentrations in the Supernatant of CFBE 41o- Cell Cultures In Vitro

**Figure 6:**
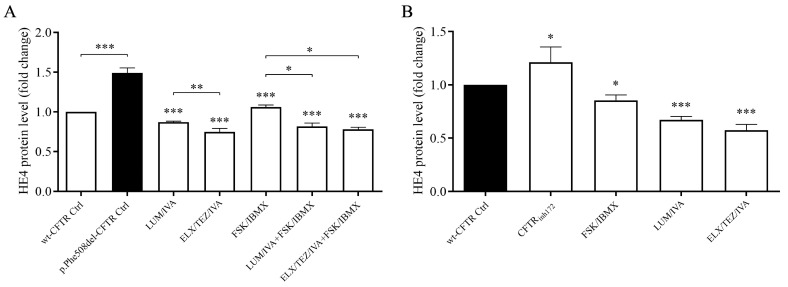
Analysis of HE4 levels in the supernatant of *p.Phe508del-CFTR* (**A**) or wt-CFTR CFBE 41o- cells (**B**) with CFTR function pharmacologically altered by ELX/TEZ/IVA or LUM/IVA treatment for 24 h. An unpaired *t*-test was performed for comparisons. Differences were considered significant at *p* < 0.05 (*), *p* < 0.01 (**), and *p* < 0.001 (***) compared to control cells.

To investigate the effects of CFTRm on HE4 protein expression, CFBE41o- cells expressing the *p.Phe508del-CFTR* variant were treated with either the triple combination of ELX/TEZ/IVA (Kaftrio^®^) or the dual combination of LUM/IVA (Orkambi^®^) for 24 h. HE4 concentrations were subsequently measured in the culture supernatants. CFBE41o- cells expressing wt-CFTR were used as controls. Consistent with previous findings [15,18], basal HE4 secretion was significantly higher in *p.Phe508del-CFTR* cells compared to wt-CFTR controls (*p* < 0.001). Both treatment regimens resulted in a marked reduction in HE4 levels compared to vehicle-treated (DMSO) control samples (*p* < 0.001 for both combinations; see Figure 6A). Importantly, ELX/TEZ/IVA produced a more pronounced decrease in HE4 concentration than LUM/IVA (*p* < 0.01). In a complementary experiment, FSK/IBMX agents known to stimulate CFTR channel activity were applied alone or in combination with CFTR modulators in *p.Phe508del-CFTR* cells. Treatment with FSK/IBMX alone led to a significant reduction in HE4 levels (*p* < 0.001), presumably through activation of residual CFTR function. Notably, co-treatment with FSK/IBMX and CFTR modulators resulted in a further decrease in HE4 expression, which was statistically significant compared to FSK/IBMX treatment alone (*p* < 0.05), reinforcing the additive effect of these compounds (Figure 6A).

To further explore the inverse relationship between CFTR function and HE4 expression, we examined the effects of CFTR inhibition in wild-type CFTR-expressing CFBE41o- cells. Pharmacological blockade using CFTR_inh172_ resulted in a statistically significant elevation of HE4 levels in the culture supernatant (*p* < 0.05), supporting the hypothesis that reduced CFTR activity contributes to increased HE4 secretion. Conversely, stimulation of CFTR function via FSK/IBMX led to a significant reduction in HE4 concentrations when compared with untreated controls (*p* < 0.05), indicating that upregulated CFTR activity exerts a suppressive effect on HE4 expression. Surprisingly, both ELX/TEZ/IVA and LUM/IVA treatments were able to reduce HE4 levels, even in CFBE41o- cells with wild-type CFTR expression. The reduction was significant in both cases (*p* < 0.001) and comparable in magnitude (Figure 6B). These data suggest that CFTR function affects basal levels of HE4 expression, and impaired function of CFTR could explain elevated HE4 concentration in CF airway epithelial cells in vitro. Taken together, these data reinforce the concept that baseline HE4 expression is modulated by CFTR activity. Impaired CFTR function, as seen in CF-derived airway epithelial cells, may underlie the elevated HE4 secretion observed in vitro. Moreover, in cells expressing the *p.Phe508del-CFTR* mutation, ELX/TEZ/IVA elicited a more substantial reduction in HE4 levels than LUM/IVA (Figure 5), consistent with its superior efficacy in rescuing CFTR function. This corresponded with a larger shift in extracellular HE4 concentration, mirroring previously reported changes in systemic HE4 levels.

## 4. Discussion

According to our current and recent studies [15,16,17,18], HE4 acts as a reliable, non-invasive, standardized, blood-based biomarker for monitoring different CFTRm therapies, as immunochemistry investigation is widely available in most routine clinical laboratories. Hence, this relatively cheap test can be easily performed during the follow-up visits of patients under still very expensive CFTRm drugs in daily practice.

To the best of our knowledge, this is the first clinical study to demonstrate a direct association between serum HE4 levels and changes in pulmonary function—specifically, delta ppFEV_1_—following treatment with triple CFTR modulator ELX/TEZ/IVA (Kaftrio^®^) in pwCF. Although the clinical benefits of this therapeutic combination have been previously assessed using a variety of parameters, including ppFEV_1_, BMI values, CFTR functional assays in both airway and the intestinal epithelia, and sweat chloride concentration in children aged 2–5 years [10] and 6–11 years [12], as well as in adolescents aged 12 years or older and adults [8,9,11,25], each of these established markers presents certain limitations. For instance, the discriminative capacity of ppFEV_1_ is diminished in patients who already exhibit moderate to near-normal lung function [26]. Furthermore, considerable variability in ppFEV_1_ has been reported due to age-related differences and variations in reference standards among populations, not to mention the technical challenges associated with performing reliable spirometry in pediatric patients [26]. Sweat chloride measurements, while frequently used as a pharmacodynamic indicator, are generally considered secondary endpoints in clinical trials. Additionally, standardization in sweat conductivity testing remains an ongoing challenge [27]. These limitations underscore the need for more accessible and robust surrogate markers, ideally from peripheral blood, that can more effectively track treatment response in clinical practice [28]. Given the chronic airway inflammation characteristic of CF, blood-based inflammatory biomarkers offer a promising route for evaluating the effectiveness of CFTRm therapies. Previous investigations have shown that serum levels of pro-inflammatory cytokines, such as interleukin-18 (IL-18) and tumor necrosis factor-α (TNF-α) exhibit a significant decline within 1 to 3 months of LUM/IVA therapy, whereas reductions in IL-1β appear to be more specific to TEZ/IVA administration [29]. In pwCF on Kaftrio^®^ therapy, the neutrophil number, fibrinogen, and CRP, along with other cytokines, such as GM-CSF, IFN-γ, IL-1α, IL-1β, IL-8, IL-12p70, IL-17A, and TNF-α, significantly decreased during a 12-month follow-up. In addition, a significant association between these inflammatory biomarkers and ppFEV_1_, BMI, and sweat chloride levels was reported [30]. Similarly, another group found that ETI reduced neutrophil counts with more mature, less inflammatory phenotypes and a shift towards an immune-resolving state associated with increased CD206 expression [31]. This CFTRm treatment also benefits liver function and nutrient absorption, improving cholesterol and albumin levels after one year of therapy [32].

Our group previously proved the utility of examining serum HE4 concentrations to assess the severity of CF lung disease in Czech and Hungarian pwCF before the administration of CFTRm [15]. HE4 levels were then efficiently utilized to monitor lung function alterations due to the efficacy of IVA in three independent cohorts of pwCF [16] and in those on Orkambi^®^ therapy [17]. CFTR dysfunction has been reported to contribute to abnormal HE4 expression via the activation of the pro-inflammatory NF-κB pathway in CF, and the effect of LUM/IVA treatment on HE4 expression has been evaluated in CFBE 41o- cells expressing *p.Phe508del-CFTR* in vitro, thereby attesting to the downregulation of the aforementioned pro-inflammatory pathway [18]. However, in vivo serum HE4 concentrations have not been studied in a large CF cohort treated with ELX/LUM/IVA thus far to assess therapeutic efficacy. Although introducing this CFTRm has led to unprecedented improvements in lung function and quality of life, along with a reduced need for lung transplantation, as reviewed in [33], the follow-up of the effectiveness of CFTRm in routine care is still necessary.

Here, we analyzed well-selected serum specimens for HE4 measurements in a sub-cohort of pwCF homozygous for the *p.Phe508del-CFTR* variant. In total, 80 pwCF on Kaftrio^®^ were enrolled from two independent national cohorts in this study, who demonstrated a substantial mean change in ppFEV1 (17.5 and 4.0%, respectively) observed from baseline to 3–6 months of therapy, similar to the results of other study populations with significant changes in the mean ppFEV1 (11%) in children [12] and adolescents (14.3%) by week 24 [8]. In cohort 1, serum HE4 levels were substantially reduced below baseline after 3 months of ETI, with a mean delta HE4 of −45.7 pmol/L, while ETI pwCF in cohort 2 showed a mean delta value of −20.7 pmol/L HE4. In parallel, pwCF on Orkambi^®^ also demonstrated reducing serum HE4 values (mean change of −18.5 pmol/L), but HE4 concentrations were significantly lower at each follow-up time point in those on ETI compared to LUM/IVA patients showing only a modest degree of lung function improvement (+1.6%), in agreement with the delta value of ppFEV1 (+2.5%) of subjects reported in a previous study under LUM/IVA with two copies of the *p.Phe508del-CFTR* variant [13]. Overall, the superior therapeutic effect of Kaftrio^®^ over Orkambi^®^ on pulmonary clinical status could be successfully monitored via serum HE4. Previously, IVA alone (Kalydeco^®^) could cause a larger alteration in plasma HE4 (−14.4 pmol/L vs−10.7 pmol/L) in a cohort with CF patients bearing at least one *p.Gly551Asp-CFTR* variant in *trans* (the Class III *CFTR* variant), since the mean delta ppFEV1 was also higher (7.0%) by 6 months of treatment in selected GOAL study participants [16] compared to PROSPECT study participants (bearing Class II *CFTR* variant p.Phe508del), with a mean delta ppFEV1 of only 2.6% [17]. Recently, Schmidt et al. also analyzed HE4 and matrix metalloproteinase 9 (MMP9) to monitor the effect of ETI, and a significant decrease in the level of both markers was observed by 6 months of ETI treatment [34]. In parallel, MMP9 was utilized in a small group of pwCF on Trikafta^®^, and plasma MMP9 expressed via the NF-κB pathway by circulating mononuclear cells was downregulated in responders. At the same time, non-responders had sustained elevated MMP9 levels under therapy, suggesting the potential role of MMP9 as a new biomarker for treatment efficacy [35].

Delta and absolute HE4 levels were statistically correlated with the delta and absolute ppFEV1 values, respectively, and a significant inverse correlation was detected between HE4 and ppFEV1 in all cohorts. Likewise, in our recent study, there was a significant negative relationship between plasma HE4 concentrations and ppFEV1 at 6 months of LUM/IVA therapy, while the mean change in HE4 from baseline similarly correlated with delta ppFEV1. When we separately analyzed this association among pwCF under 18 years of age, a stronger correlation was found in childhood [17].

The discriminatory “power” of the mean change in serum HE4 was determined by ROC-curve analysis at the time of lung function improvement (i.e., 4% by 3–6 months of treatment). The mean change in HE4 demonstrated a substantial AUC value in both cohort 1 (0.765) and cohort 2 (0.789) on ETI and, based on age, in children (0.791) and adults (0.788) on ETI. A similar AUC value of delta HE4 (0.806) was recently found, especially after 1–2 months of IVA medication, with 81% sensitivity and 89% specificity [16]. When 5% of delta ppFEV1 became the classifier in a large group of LUM/IVA subjects, the AUC value was 0.678 in all pwCF, while it was higher in children (0.791) [17].

The rescue of CFTR function by CFTRm was observed using the whole-cell patch-clamp technique in the current study, similar to previous publications [18,23]. Based on our current data, we considered *p.Phe508del-CFTR* function rescued, since Boinot et al. suggested that if the current density is greater than the threshold of 4 pA/pF after treatment with CFTRm, the CFTR activity be considered to be corrected [23]. Consequently, HE4 was significantly reduced by both the ELX/TEZ/IVA and LUM/IVA combinations. Importantly, ELX/TEZ/IVA caused a more substantial alteration in the Cl^−^ current and the HE4 concentrations than LUM/IVA. This observation was corroborated by our recent in vitro experiments in CFBE 41o- cells expressing *p.Phe508del-CFTR*, where treatment of these cells with both TEZ/IVA and LUM/IVA showed significantly decreased HE4 expression compared to untreated cells. Still, overall HE4 concentrations did not normalize [18]. All these data underline that HE4 levels do not reach normal (i.e., “wild-type levels”) in pwCF, even in the presence of improved CFTR function following administration of CFTRm. This finding is consistent with the partial correction of *p.Phe508del-CFTR* by CFTRm, as studies have shown that ETI therapy restores CFTR function in nasal and intestinal epithelia to ~40–50% of CFTR function in healthy people [25,36,37]. In summary, ETI caused a larger reduction in HE4 expression in the cell cultures in vitro, correlating with altered serum HE4 levels observed in ex vivo samples.

This manuscript has some limitations. First, we analyzed the serum HE4 concentration under up to 6 months of medication, and no further follow-up samples were available to monitor the long-term effects of these CFTRm agents via HE4 levels. Secondly, the mechanical relationship between the improvement in CFTR function by CFTRm treatment and the expression of HE4 was not fully investigated in CFBE cells; thus, additional ex vivo and in vitro studies are required.

## 5. Conclusions

Serum HE4 negatively correlates with lung function improvement and monitors better drug efficacy in pwCF under ETI than LUM/IVA. In addition, ETI caused a larger reduction in HE4 expression vs. LUM/IVA in CFBE cell cultures in vitro, in agreement with altered serum HE4 levels observed in ex vivo CF samples in response to these drugs.

## Figures and Tables

**Table 1:** Main demographic and clinical parameters of all study participants. * denotes a treatment duration of 3 months instead of 6 months.

Characteristics	CF Cohort 1 on Kaftrio^®^ (n = 51)	CF Cohort 2 on Kaftrio^®^ (n = 29)	CF Cohort on Orkambi^®^ (n = 43)
Age (years) (median, min–max)	27 (20–47)	12 (6–50)	14 (11–53)
Gender (f/m), n	28/23	17/12	23/20
Baseline sweat chloride (mmol/L) (median, min–max)	103 (61–1 19)	112 (66–149)	128 (61–166)
Baseline ppFEV1 (%) (median, min–max)	73 (24–107)	89 (40–147)	70 (23–120)
Pre-treatment BMI (kg/m^2^) (median, min–max)	21.9 (17.0–34.4)	16.3 (13.2–25.3)	17.9 (13.2–27.9)
Change in ppFEV1 by 3 * or 6 m (%) (mean, 95% CI)	17.5 (14.5 to 20.5) *	4.0 (−3.9 to 12.1)	1.6 (−1.6 to 4.7)
Baseline serum C-reactive protein (mg/L) (median, min–max)	4.5 (0.5–43.4)	2.4 (0.2–96.4)	2.3 (0.1–57.4)
Baseline serum creatinine (µmol/L) (median, min–max)	-	71 (32–96)	68 (32–107)
Bacterial colonization (y/n), n	-	7/22	10/33
Responders/non-responders, n	46/5	19/10	22/21

## Data Availability

Data sharing does not apply to this article.

## Supplementary Materials

The following supporting information can be downloaded at: https://www.mdpi.com/article/10.3390/jcm14176188/s1. Figure S1: Determination of the discriminatory power of delta HE4 in Orkambi^®^-treated pwCF (A), and that of absolute HE4 if 4.0% of delta ppFEV1 at 3 or 6 months of Kaftrio^®^ (B) or Orkambi^®^ (C) treatment was used as the binary classifier using ROC-AUC curve analyses.

## Abbreviations

The following abbreviations are used in this manuscript:

HE4	human epididymis protein 4
pwCF	patients with CF
CFTRm	CFTR modulator
CFTR	CF transmembrane conductance regulator
CFBE	cystic fibrosis bronchial epithelial cell
LUM/IVA	lumacaftor/ivacaftor
ETI	elexacaftor/tezacaftor/ivacaftor
ppFEV1	FEV1% predicted value
CFTRm	CFTR modulators
CRP	C-reactive protein
FSK	Forskolin
IBMX	3-isobutyl-1-methylxanthine
DMSO	dimethyl sulfoxide
ROC	the receiver operating characteristic curve
AUC	area under the curve
IL-18	interleukin-18
TNF-α	tumor necrosis factor-α
