# Supplementary material for: Comparison of the Effect of CFTR Modulators elexacaftor/tezacaftor/ivacaftor and lumacaftor/ivacaftor via Serum Human Epididymis Protein 4 Concentration in p.Phe508del-CFTR Homozygous Cystic Fibrosis Patients
Source: J Clin Med. 2025 Sep 2;14(17):6188. doi: 10.3390/jcm14176188 (PMC12429638; doi:10.3390/jcm14176188)
Supplement: Supplementary file 1 [file jcm-14-06188-s001.zip › jcm-3832853-supplementary material.pdf]

Journal of
Clinical Medicine

MDPI

Supplementary Figure S1.

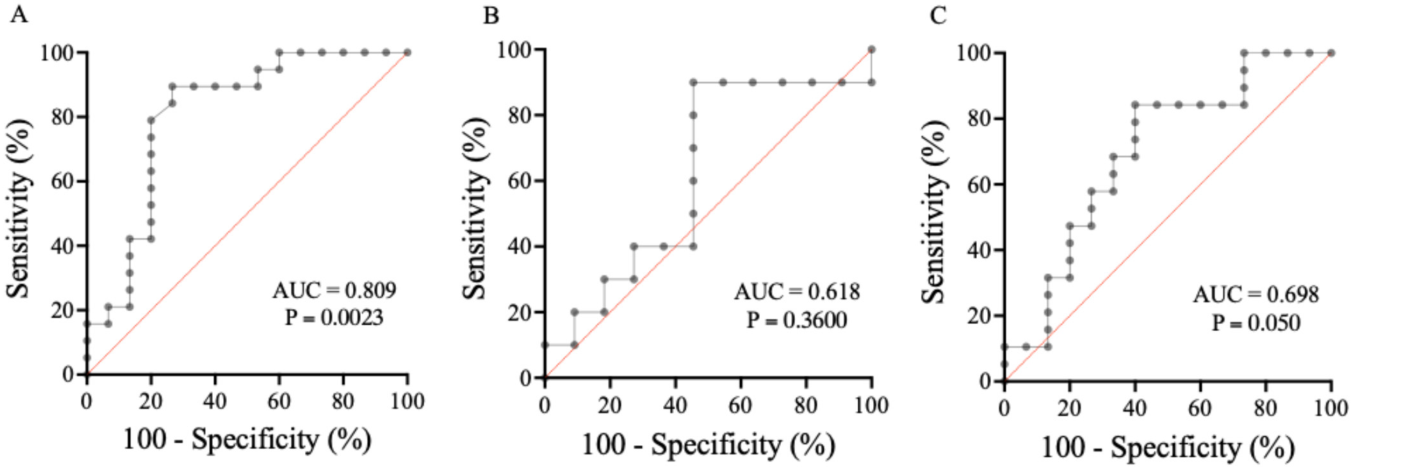

Figure S1: Determination of the discriminatory power of delta HE4 in Orkambi®-treated pwCF (A), and that of absolute HE4 if 4.0% of delta ppFEV1 at 3 or 6 months of Kaftrio® (B) or Orkambi® (C) treatment was used as the binary classifier using ROC-AUC curve analyses.
